# Association of Systemic Inflammatory and Immune Indices With Survival in Canine Patients With Oral Melanoma, Treated With Experimental Immunotherapy Alone or Experimental Immunotherapy Plus Metronomic Chemotherapy

**DOI:** 10.3389/fvets.2022.888411

**Published:** 2022-07-06

**Authors:** Jéssica Soares Garcia, Victor Nowosh, Rossana Verónica Mendoza López, Cristina de Oliveira Massoco

**Affiliations:** ^1^Department of Pathology, School of Veterinary Medicine and Animal Science, University of São Paulo, São Paulo, Brazil; ^2^Center for Translational Research in Oncology of the Institute of Cancer of São Paulo State, São Paulo, Brazil

**Keywords:** neoplasm, cancer vaccines, inflammation, immunosuppression, immunomodulation

## Abstract

Analysis of the expression of inflammatory markers before starting treatment in human patients with cancer helps to predict outcomes and prognosis; however, there have been few studies on this topic in veterinary medicine. The present study aimed to evaluate inflammatory indices before treatment with autologous antitumor vaccine alone or this vaccine plus metronomic chemotherapy (MC) to predict response and prognosis. The indices included the neutrophil–lymphocyte ratio (NRL), platelet–lymphocyte ratio (PLR), monocyte–lymphocyte ratio (MLR), systemic immune-inflammation index (SII), C-reactive-protein–albumin ratio (CRP/ALB), lactate dehydrogenase level (LDH), frequency of blood lymphocyte subsets (CD4^+^, CD8^+^, Treg, and CD4/CD8 ratio) and frequency of blood myeloid-derived suppressor cells (MDSCs: monocytic [M]- MDSCs, and granulocytic [PMN]-MDSCs). Blood samples were collected from 25 dogs with oral melanoma treated with the autologous antitumor vaccine and from nine dogs that received MC plus vaccine before surgery. There were no statistically significant differences in the progression-free survival (PFS) or overall survival (OS) between the groups. In addition to the clinical stage, the CRP/ALB ratio and blood circulating Tregs in the univariate analysis showed an association with PFS and OS, and thus were selected for multivariable analysis. The CRP/ALB ratio was associated with PFS [hazard ratio (HR), 1.1; 95% confidence interval (CI), 1.0–1.1; *p* = 0.017] and OS [HR, 1; 95%CI, 1.0–1.1; *p* = 0.023]. Similarly, Treg was associated with PFS (HR, 1.6; 95% CI, 1.2–2.1; *p* = 0.001) and OS (HR, 1.6; 95% CI, 1.2–2.1; *p* = 0.001). Furthermore, canine patients with a CRP/ALB ratio above the cut-off point of 1.9 (established by receiver operating characteristic curve analysis) had worse PFS and OS, indicating the impact of the preoperative CRP/ALB ratio on the PFS and OS of dogs with oral melanoma. The CRP/ALB ratio and frequency of circulating Tregs are potential prognostic markers in dogs with oral melanoma.

## Introduction

Prognostic and predictive markers for companion animal neoplasias are gaining increasing interest in veterinary medicine, particularly for heterogeneous neoplasias, such as canine oral melanoma. Assessing the tumor microenvironment characteristics and the immune/inflammatory profile are known to improve prognostic precision and refine the predicted response for personalized therapy approaches.

Currently, immunotherapy in veterinary patients has attracted interest in experimental and clinical trials. The growing use of immunotherapy for the treatment of different types of cancer in human medicine and the marked variation in response to this therapeutic approach have motivated the study of blood markers that can predict the patient's response to therapy, not only in the search for prognostic markers, but also to select patients appropriately, because this therapeutic modality is costly (1–3).

Canine oral melanoma is the most frequent neoplasm of the oral cavity in dogs and has an invasive and highly metastatic nature (4, 5). Treatment is based on surgical removal of the neoplasm, with or without radiotherapy and electrochemotherapy. After local control, the use of adjuvant therapy is recommended for metastasis control (4, 5). This neoplasm is difficult to control, and consequently there have been numerous studies using different modalities of immunotherapy, and studies attempting to predict responses to such treatment. In addition to predicting treatment response, determining prognostic factors could add parameters and improve the accuracy of the clinical staging in patients. The possibility of genetically engineering immunogenic antigens for melanoma vaccines (6), which could be financially limited for clinical use in domestic animal data from animal models, supports the potential use of active immunotherapy using tumor lysate vaccines with pathogen adjuvants, such as Bacillus Calmette–Guerin (BCG), to improve treatment for melanoma (7, 8).

Inflammation contributes to cancer progression, since the continuous presence of inflammatory cells creates the conditions for tumor development, e.g., apoptosis evasion, sustained angiogenesis, self-sufficiency in growth signaling, insensitivity to anti-growth signaling, and tissue invasion/metastasis. Infiltration of immune system cells into the tumor microenvironment contributes to cancer progression (9, 10). The chronic systemic inflammatory response in human patients with cancer (hepatocellular carcinoma, colorectal carcinoma, and gastric carcinoma) is believed to be directly related to the degree of disease progression (11–13). Considering the cost/benefit ratio in a veterinary setting, some inflammatory parameters, calculated based on the blood cell count, serum lactate dehydrogenase (LDH) measurement, and blood immunophenotyping are not costly, and thus are applicable in clinical practice.

Some useful indices are calculated based on blood cell count and inflammatory proteins, such as the neutrophil–lymphocyte ratio (NLR), platelet–lymphocyte ratio (PLR), monocyte–lymphocyte ratio (MLR), systemic immune-inflammation index (SII), C-reactive-protein–albumin ratio (CRP/ALB ratio), and frequency of circulating immune cells (subsets of T lymphocytes and myeloid-derived suppressor cells). Inflammatory indices, widely studied in human cancer patients with melanoma and different types of carcinomas (colorectal, cervical, urothelial, pancreatic, and nasopharyngeal), are useful in indicating prognosis and predicting responses to different therapeutic modalities, such as surgery, chemotherapy, immunotherapy, and specific antitumor drugs (1–3, 12, 14–19).

High values of these indices or a high frequency of immunosuppressive cells are found in human patients with more aggressive cancers and more advanced stages of the disease (20–22). However, there have been few relevant studies in veterinary medicine (23–30). For human patients with melanoma, in addition to the immune profile, the serum level of LDH is considered an important prognostic factor (31), particularly in patients with metastases; however, in veterinary medicine, there have been no studies in dogs with oral melanoma, although there are some reports on other neoplasms (32–34).

This study aimed to assess whether the values of inflammatory indices, serum LDH levels, and frequency of immune circulating cells could predict the response to treatment and prognosis that correlate with overall survival (OS) and progression-free survival (PFS) in dogs with oral melanoma that received tumor lysate vaccine with BCG [alone, or in combination with metronomic chemotherapy (MC)].

## Materials and Methods

### Patient Enrollment

Blood, tumor samples, and clinical data were prospectively collected from 90 canine patients with oral melanoma attending several Brazilian private clinics and teaching veterinary hospitals. Of these, 25 patients were included in this study. Sixteen received only the tumor lysate vaccine as adjuvant treatment, and nine received the tumor lysate vaccine plus MC. Surgery [complete or incomplete excision alone or plus electrochemotherapy (ECT)], adjuvant treatment (tumor lysate vaccine alone or plus MC), and follow-up were performed in the respective veterinary clinics (Table 1). The clinical study was neither randomized nor blinded, and the treatment was chosen by a clinical veterinarian in conjuntion with the patient's owner.

**Table 1:** Characterization and treatment of 25 dogs with oral melanoma.

**ID**	**Breed**	**Sex**	**Age (years)**	**Clinical Stage**	**Surgery**	**ECT**	**MC**
1	Golden Retriever	Male	12	III	Incomplete	Yes	Not
2	Dachshund	Female	14	IV	Incomplete	Yes	Not
3	Golden Retriever	Male	9	IV	Incomplete	Yes	Yes
4	Mixed	Female	16	IV	Incomplete	Yes	Not
5	Schnauzer	Male	11	II	Complete	Not	Not
6	Shih Tzu	Male	15	II	Incomplete	Yes	Yes
7	Mixed	Male	15	III	Incomplete	Yes	Not
8	Mixed	Female	7	III	Complete	Not	Yes
9	Mixed	Female	13	III	Incomplete	Not	Not
10	Mixed	Female	16	III	Incomplete	Yes	Not
11	Poodle	Male	17	II	Incomplete	Not	Not
12	Boxer	Male	11	II	Incomplete	Yes	Not
13	Rottweiler	Male	8	III	Incomplete	Yes	Not
14	Mixed	Female	12	II	Complete	Not	Not
15	Pinscher	Male	13	II	Complete	Yes	Not
16	Beagle	Male	13	IV	Incomplete	Yes	Yes
17	Dachshund	Female	17	II	Incomplete	Yes	Not
18	Cocker Spainel	Male	14	III	Incomplete	Yes	Yes
19	Dachshund	Male	13	III	Complete	Yes	Yes
20	Pinscher	Female	11	III	Complete	Yes	Not
21	Yorkshire	Female	15	III	Incomplete	Not	Yes
22	Dachshund	Male	14	III	Incomplete	Yes	Not
23	Mixed	Male	15	III	Incomplete	Yes	Not
24	Chow Chow	Male	13	III	Incomplete	Not	Yes
25	Mixed	Male	16	III	Incomplete	Yes	Yes

*ECT, electrochemotherapy; MC, metronomic chemotherapy*.

The vaccine was produced in the laboratory of the Department of Pathology (School of Veterinary Medicine and Animal Science, University of São Paulo) and delivered to veterinarians. The study was approved by the Ethics Committee on Animal Use (CUSA/FMVZ-USP) under protocol number 3716290817, and all animals were included in the study after obtaining their owners' consent.

### Inclusion and Exclusion Criteria

Canine patients with oral melanoma diagnosed by histopathological and immunohistochemical analyses of amelanotic samples were included. Immunohistochemical antibodies used to confirm melanoma were directed against HMB-45, tyrosinase, and Melan-A present in the melanoma cocktail (Bio SB, Santa Barbara, CA, USA).

Dogs without previous treatment or who had completed previous treatment 1 month earlier were included. Patients with concomitant diseases, such as advanced heart disease, advanced chronic kidney disease, endocrine disorders, infectious diseases, concomitant neoplasms, missing data, or death from melanoma-unrelated causes were excluded (35, 36).

### Treatment Strategies, Evaluation, and Disease Assessment

All patients underwent surgical removal of the oral melanoma; the excision was complete in some and incomplete in others. Additionally, ECT was performed in some patients to expand the surgical bed. ECT was applied to the surgical bed immediately after incomplete tumor excision and was performed by trained professionals. It involved the application of 15,000 UI/m^2^ intravenous bleomycin, followed 5 min later by eight square-wave electric pulses of 1,220 V/cm at an interval of 100 mHz to 1 Hz. The electroporators used by different oncology veterinary services were a Vetpulser BK 100 (Brunner, São Paulo, Brazil), VetCP 125 (Veterinary Cell Porator, São Paulo, Brazil), and ELECTROvet EZ (Leroy Biotech, Saint-Orens-de-Gameville, France).

The World Health Organization classification of tumors in domestic animals was used for clinical staging, and examinations were performed before surgery and every 3 months thereafter, or earlier if necessary (37). The examinations included physical examination, complete blood count, complete serum biochemistry, triple thoracic radiography, abdominal ultrasound examination, and echocardiography. Bone invasion data were unavailable. MC consisted of oral cyclophosphamide 15 mg/m^2^ daily or every other day.

**Figure 1:**
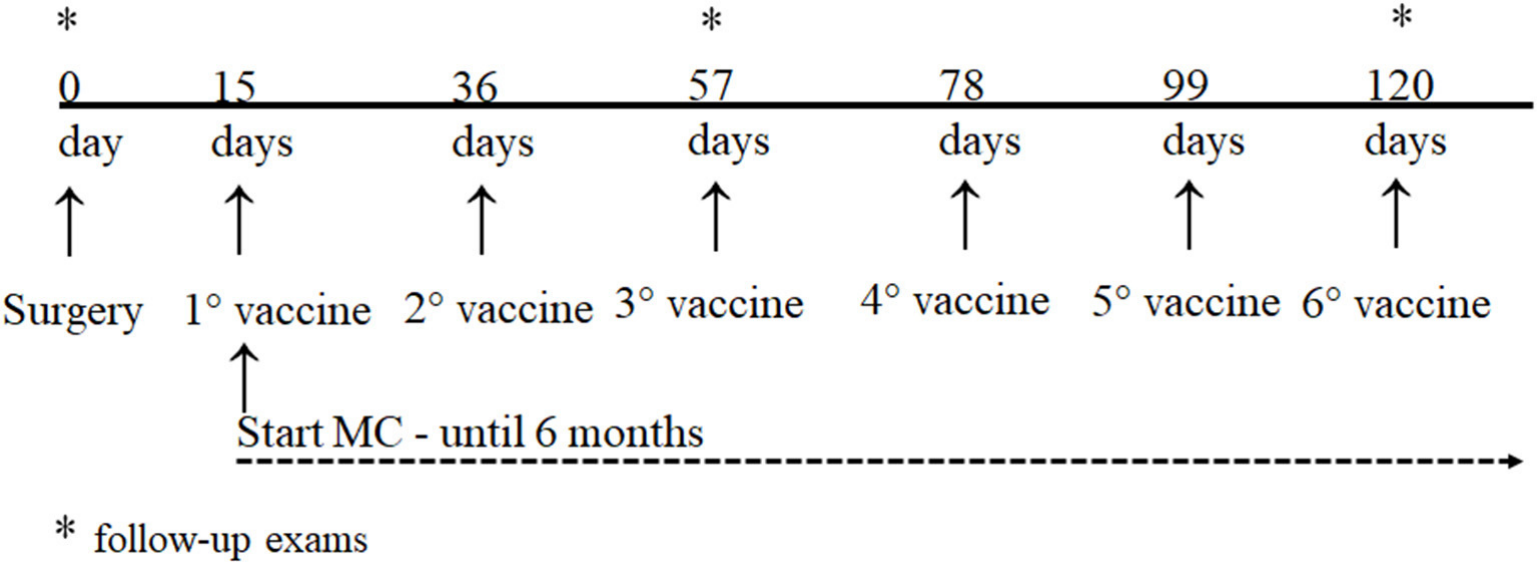
Treatment and follow-up protocol for dogs with oral melanoma.

The tumor lysate vaccine with BCG is an autologous vaccine, as it is prepared with the patient's tumor (each dose contained 5 × 10^6^ to 9 × 10^6^ tumor cells). The complete vaccine protocol is presented in the Supplementary Material. The first dose was administered 15 days after surgery, and the following doses were administered at intervals of 21–28 days. A minimum of 3–6 doses is recommended. The vaccine was administered intradermally near the popliteal lymph node (distant from the tumor). Patients undergoing concomitant use of MC started the first dose of the vaccine for a minimum period of 6 months (Figure 1).

The primary outcome was PFS, which was defined as the time interval between the surgical removal of the tumor (date of inclusion in the study) and detection of metastasis and/or local recurrence (38). The patients were examined every 21 or 28 days after they were vaccinated, and every 3 months after the end of the vaccine treatment. At the same time, imaging and blood tests were performed. The secondary outcome evaluated was OS, which was defined as the duration in days between the surgical removal of the tumor and death or the last follow-up.

### Blood Harvest and Inflammatory Indices Analysis

Blood samples (5 ml) were collected from the cephalic or jugular vein and were stored in EDTA anticoagulant tubes. Absolute red blood cell, leukocyte, and platelet counts were determined using a Poch-100Iv Diff (Sysmex, São Paulo, Brazil) veterinary automatic cell counter, whereas differential leukocyte counts and the morphological analysis of cells were obtained and performed, respectively, by two different and experienced pathologist veterinarians, under a microscope, using a freshly stained Rosenfeld slide.

An average of blood count data was used to calculate the inflammatory indices: NRL (n° neutrophils/n° lymphocytes), PLR (n° platelets/n° lymphocytes), MLR (n° monocytes/n° lymphocytes), and SII (n° platelets × NRL).

Blood was stored in a tube (with or without clot activator), and serum was separated up to 24 h after collection and stored at −80°C until use. LDH (kinetic determination) and albumin (bromocresol green) were measured using an automated device (Chem Well T; Labtest Interteck, Minas Gerais, Brazil) with reagents supplied by the manufacturer. CRP levels were determined using a chemistry immunoassay technique (Catalyst One, Idexx, São Paulo, Brazil). The CRP/ALB ratio was calculated by dividing the CRP by the albumin value.

### Peripheral Blood Mononuclear Cells Isolation and Immunophenotyping by Flow Cytometry

The remaining blood used for the inflammatory index was used for PBMC separation using the Ficoll-Paque density gradient centrifugation method (at 900 g for 25 min, break-off, 22 °C; Sigma cat# 15120), and cells obtained after separation were cryopreserved and stored in liquid nitrogen until analysis. PBMCs were thawed at 37°C, and nonspecific antibody binding was blocked by the pretreatment of cells with Fc-block reagent (eBiosciences, Thermo Fisher Scientific, Waltham, MA, USA, mixed by vortexing, and incubated for 15 min at 25°C.

For the frequency of lymphocyte subsets, aliquots of 1 × 10^5^ cells in a final volume of 100 μl were first incubated with an FcR blocking reagent (eBiosciences, Thermo Fisher Scientific, Waltham, MA, USA), vortexed, and incubated for 15 min at 25 °C. Different antibodies were used for the detection of the T cell population, and the following combinations of monoclonal antibodies (MAb) were used: anti-CD3-FITC/anti-CD4-APC/anti-CD8-Alexa Fluor 647 (cat #TC014 Bio-Rad, Hercules, CA, USA; CA clone 17.2a12; clone YKIX 302.9; and clone YCATE 55.9, respectively) for CD4 and CD8 T lymphocyte labeling, and a combination of anti-CD4-APC antibody (cat# MCA1038GA Bio-Rad; clone YKIX 302.9), anti-CD25-FITC (cat# MCA5916 Bio-Rad, clone BC96), and anti-Foxp3-RPE (cat# 72-5774-40 eBiosciences, Thermo Fisher Scientific, clone 236A/E7) antibody for Treg lymphocytes detection. Incubation was performed for 30 min at 4°C and protected from light. For lymphocyte staining, after following the surface staining protocol with CD4 and CD25 antibodies, lymphocytes cells were incubated with fixation/permeabilization buffer (cat# 00-5123-43 eBioscience, Thermo Fisher Scientific) for 20 min at 4°C in the dark. After fixation, cells were washed with 2 ml of permeabilization buffer, and the anti-Foxp3 antibody was added (1:50) in 100 μl of permeabilization buffer to stain intracellular markers, incubated for 20 min at 25°C, and protected from light. After incubation, cells were washed twice with 2 ml of permeabilization buffer and then washed once with 1 ml of phosphate-buffered saline containing 2% fetal calf serum (cat# ES-009-B, Merck).

MDSCs represent a heterogeneous cell population consisting of two major subsets: monocytic (M) and granulocytic (PMN) MDSCs, which are identified by a combination of multiple lineage markers. Another aliquot of PBMCs was thawed and diluted to 1 × 10^5^ cells in a final volume of 100 μl and was incubated with Fc blocking as previously described. The (M)-MDSCs population was identified as CD11b+CD14-MHCII-, while the (PMN)-MDSCs were CD11b+CD14+MHCII- (PE/Cy5 anti-mouse CD11b, cat# MCA177S Abcam, Waltham, MA, USA, clone CA16.3E10), CD14 (mouse anti-human CD14 Alexa Fluor 647, cat# MCA1568A647 Bio-Rad/, and MHCII (rat anti-dog MHC CLASS II FITC, cat# MCA1044F Bio-Rad, clone YKIX334.2). After washing, all sets of cells were fixed with phosphate-buffered saline containing 1% paraformaldehyde, acquired using a FACSCalibur flow cytometer (BD Biosciences, Franklin Lakes, NJ, USA), and analyzed for cell surface phenotypes using FlowJo software (Tree Star, Bioz, Los Altos, CA, USA). For each sample, multiparametric data were acquired for 10,000 events. The percentage of MDSCs was calculated based on the percentage of cells labeled within the overall PBMC population. Appropriate isotype control was also used (data not shown). During data acquisition, a gate was drawn to exclude debris cells and lymphocytes, and myeloid cells were identified based on the cell characteristic properties in the forward (FSC) and side (SSC) scatter.

### Statistical Analysis

The log-rank test was used to compare PFS and OS between dogs that received only the tumor lysate vaccine and those that received the lysate vaccine in combination with MC. Cox regression was performed to calculate the hazard ratio (HR) with a 95% confidence interval (CI) for clinical stage, inflammatory indices (NRL, MLR, PLR, SII, and CRP/ALB ratio), LDH dosage, and the frequency of circulating cells (lymphocytes CD4+, CD8+, CD4/CD8 ratio, Tregs, (M)-MDSCs, and (PMN)-MDSCs) to select variables for inclusion in multivariate analysis. Consequently, another Cox regression analysis with the selected variables was performed to generate multiple models. The CRP/ALB ratio was used to construct a receiver operating characteristic (ROC) curve and establish a cut-off value that was then submitted to the log-rank test to compare PFS and OS between groups. Statistical analyses were performed with SPSS version 25.0 (IBM Corp., Armonk, NY, USA), and significance was set at 5%.

## Results

Of the 25 included patients, 16 (64%) were male and 9 (36%) were female, with a mean age of 13 years (standard deviation, 2.6). The most affected breeds were mixed breeds [8 (32%)], Dachshund [4 (16%)], Pinscher [2 (8%)], Golden Retriever [2 (8%)], Poodle [1 (4%)], Cocker Spaniel [1 (4%)], Yorkshire [1 (4%)], Rottweiler [1 (4%)], Schnauzer [1 (4%)], Boxer [1 (4%)], Shih Tzu [1 (4%)], Chow Chow [1 (4%)], and Beagle [1 (4%)]. Regarding clinical staging, 7 (28%), 14 (56%), and 4 (16%) patients were diagnosed with stage II, III, and IV cancers, respectively; all stage IV cancer patients had lung metastasis. Of the 18 patients with stage III and IV cancer, 12 (67%) showed lymph node metastasis at the time of inclusion. Furthermore, 21 (84%) and 4 (16%) patients had melanotic and amelanotic melanomas, respectively.

Complete and incomplete surgical excision of the tumor was performed in six (24%) and 19 (76%) patients, respectively. ECT was performed in 18 (72%) patients immediately after surgical excision and was not performed in seven (28%) patients. Furthermore, nine (36%) patients received MC.

First, a log-rank test was conducted to evaluate patients who received MC in addition to the vaccine for PFS and OS. There were no statistically significant differences between the two groups in terms of PFS (*p* = 0.294) or OS (*p* = 0.553). The median, minimum, and maximum values of inflammatory indices, LDH level, CRP, albumin, and frequency of circulating cells according to the clinical stage are summarized in Table 2.

**Table 2:** Median, minimum, and maximum value of inflammatory indices (NRL, PLR, MLR, SII, and CRP/ALB ratio), CRP, albumin, LDH, and frequency of circulating cells (lymphocytes CD4^+^, CD8^+^, CD4/CD8 ratio, Tregs, (M)-MDSCs, and (PMN)-MDSCs) according to clinical stage.

**Clinical stage**	**All dogs (*n* = 25)**	**II (*n* = 7)**	**III (*n* = 14)**	**IV (*n* = 4)**
NRL	5.3 (1.6–20.3)	5 (1.6–6.2)	5.1 (1.7–20.3)	9.3 (5.1–12.8)
PLR	250 (67–581)	267 (136–481)	249 (66–581)	254 (93–467)
MLR	0.3 (0.1–1.0)	0.3 (0.1–0.7)	0.3 (0.1–0.7)	0.5 (0.2–1.0)
SII (× 10^9^/L)	2,430 (488–7,117)	1,960 (1,096–4,648)	2,160 (488–7,117)	5,438 (3,164–6,284)
CRP (mg/L)	18 (0.4–86)	7 (0.5–65)	18 (3–62)	40 (2–8.6)
Albumin (g/dl)	3.1 (1.8–4.9)	3 (2.7–4.9)	3.1 (2.2–3.8)	3.2 (1.8–3.8)
CRP/ALB ratio	5.3 (0.1–42.2)	2.1 (0.1–24.1)	5.5 (0.9–21.4)	14 (0.6–42.2)
LDH (U/L)	762 (95–2,982)	666 (514–2,982)	865 (95–2,273)	791 (400–1,582)
CD4 (%)	43.5 (15–65)	43.5 (15–57)	45.7 (29.5–65)	37.5 (29–47)
CD8 (%)	26.7 (6–45)	28.7 (6–45)	28 (10–44)	21.5 (14–26.7)
CD4/CD8 ratio	1.5 (0.34–9.17)	1.2 (0.3–9.1)	1.5 (1.0–5.4)	0.3 (1.4–2.7)
T reg (%)	2.7 (0.3–9.7)	2.7 (0.3–3)	3 (0.8–9.7)	3 (0.5–4)
(M)-MDSCs (%)	10.8 (2–29.2)	10.7 (4.8–29.2)	14.9 (8–28)	5.5 (2–25)
(PMN)-MDSCs (%)	12 (3.9–34)	7.5 (3.9–34)	12.2 (4–34)	12.5 (9–19)
Total MDSCs (%)	22.8 (2–34)	18.2 (3.9–34)	27.1 (4–34)	18 (2–25)

*NRL, neutrophil–lymphocyte ratio; PLR, platelet–lymphocyte ratio; MLR, monocyte–lymphocyte ratio; SII, systemic immune–inflammation index; CRP, C-reactive protein; CRP/ALB ratio, C-reactive protein–albumin ratio; LDH, lactate dehydrogenase; Treg, T regulatory cells; (M)-MDSCs, monocytic myeloid-derived suppressors cells; (PMN)-MDSCs, granulocytic myeloid-derived suppressors cells*.

Univariate analysis (Cox regression model) was performed for each variable in relation to PFS and OS. In addition to the previously noted variables, the clinical stage and type of surgery performed (complete or incomplete) were identified, which have previously been recognized as prognostic factors in dogs with oral melanoma (Table 3).

**Table 3:** Effect of clinical stage, surgical margins, adjuvant treatment (tumor lysate vaccine alone or plus MC), inflammatory indices (NRL, PLR, MLR, SII, and CRP/ALB ratio), albumin, LDH, and frequency of circulating cells (lymphocytes CD4, CD8, CD4/CD8 ratio, Tregs, (M)-MDSCs, and (PMN)-MDSCs) on the PFS and OS of 25 canine patients with oral melanoma.

**Variable**	**PFS^**a**^**	**HR (95% CI)**	***p*–value**	**OS^**a**^**	**HR (95% CI)**	***p*–value**
All dogs (*n* = 25)	68			115		
Stage						
II (*n* = 7)	181	1	0.015*	265	1	0.043*
III (*n* = 14)	40	4.0 (1.4–11.6)	0.011*	101	2.9 (0.9–8.4)	0.051*
IV (*n* = 4)	38	7.1 (1.7–30.3)	0.008*	68	5.8 (1.4–24.4)	0.015*
Surgery						
Incomplete (*n* = 19)	56	1		94	1	
Complete (*n* = 6)	121	1.6 (0.6–4.4)	0.346	153	1.3 (0.5–3.5)	0.635
Adjuvant						
LyVaccine + MC (*n* = 9)	56	1		115	1	
LyVaccine only (*n* = 16)	78	1.6 (0.7–3.7)	0.298	134	1.4 (0.6–3.3)	0.459
NRL		1.0 (0.9–1.1)	0.333		1.0 (0.9–1.1)	0.369
PLR		0.9 (0.9–1.0)	0.412		0.9 (0.9–1.0)	0.621
MLR		2.6 (0.4–17.9)	0.329		4.4 (0.6–30.4)	0.127
SII		1.0 (1.0–1.0)	0.365		1.0 (1.0–1.0)	0.301
CRP		1.0 (0.99–1.0	0.064		1.0 (0.9–1.0)	0.094
Albumin		0.5 (0.2–1.1)	0.072		0.4 (0.1–1.0)	0.058
CRP/ALB ratio		1.0 (1.0–1.1)	0.053*		1.0 (0.9–1.1)	0.060
LDH		1.0 (0.9–1.0)	0.138		1.0 (0.9–1.0)	0.249
CD4		0.9 (0.9–1.0)	0.248		0.9 (0.9–1.0)	0.153
CD8		1.0 (0.9–1.0)	0.846		1.1 (0.9–1.0)	0.596
CD4/CD8 ratio		0.9 (0.7–1.2)	0.580		0.9 (0.7–1.2)	0.441
Treg		1.5 (1.2–1.9)	0.001*		1.4 (1.1–1.7)	0.001*
(M)–MDSCs		1.0 (0.9–1.1)	0.311		1.0 (0.9–1.1)	0.490
(PMN)–MDSCs		1.0 (0.9–1.1)	0.666		0.9 (0.9–1.0)	0.793

^a^Median in days; *Statistical significance.

*95%CI, 95% confidence interval; HR, hazard ratio; OS, overall survival; PFS, progression–free survival; LyVaccine, lysate vaccine plus BCG; MC, metronomic chemotherapy; NRL, neutrophil–lymphocyte ratio; PLR, platelet–lymphocyte ratio; MLR, monocyte–lymphocyte ratio; SII, systemic immune-inflammation index; CRP, C-reactive-protein; CRP/ALB ratio, C-reactive-protein-albumin ratio; LDH, lactate dehydrogenase; Treg, T regulatory cells; (M)-MDSCs, monocytic myeloid-derived suppressors cells; (PMN)- MDSCs, granulocytic myeloid-derived suppressors cells*.

Based on the results of the univariate analysis, the clinical stage, CRP/ALB ratio, and Treg frequency showed a statistically significant difference in relation to PFS and OS and were selected for multivariable analysis (Table 4).

**Table 4:** Effect of clinical stage, surgical margins, inflammatory indices (NRL, PLR, MLR, SII, and CRP/ALB ratio), albumin, LDH, and frequency of circulating cells (lymphocytes CD4, CD8, CD4/CD8 ratio, Tregs, (M)-MDSCs, and (PMN)-MDSCs) on the PFS and OS of 25 canine patients with oral melanoma.

**Variable**	**PFS^**a**^**	**HR (95% CI)**	***p*–value**	**OS^**a**^**	**HR (95% CI)**	***p*–value**
All dogs	68			115		
Stage						
II	181	1	0.010*	265	1	0.015*
III	40	4.0 (1.3–12.6)	0.017*	101	3.1 (0.9–10.1)	0.054*
IV	38	10.2 (2.1–50.5)	0.004*	68	12.2 (2.2–67.5)	0.004*
CRP/ALB ratio		1.1 (1.0–1.1)	0.017*		1.0 (1.0–1.1)	0.023*
Treg		1.6 (1.2–2.1)	0.001*		1.6 (1.2–2.1)	0.001*

^a^Median in days; *Statistical significance.

*95%CI, 95% confidence interval; HR, hazard ratio; OS, overall survival; PFS, progression-free survival; CRP/ALB ratio, C-reactive protein/Albumin ratio; Treg, T regulatory cells*.

After the multivariable analysis showed a statistically significant difference in clinical staging, the CRP/ALB ratio and Treg frequency were subjected to ROC curve analysis, in relation to PFS and OS. Analysis of the ROC curve for Treg showed an AUC of 0.424 for PFS and 0.523 for OS; thus, it was not possible to establish a cut-off point. The analysis of the ROC curve for the CRP/ALB ratio showed an AUC of 0.891 for PFS and 0.886 for OS, and a cut-off score of 1.9 was selected. This cut-off score had a sensitivity of 78.3% and 81.8%, a specificity of 100% and 100% for predicting PFS and OS, respectively.

**Figure 2:**
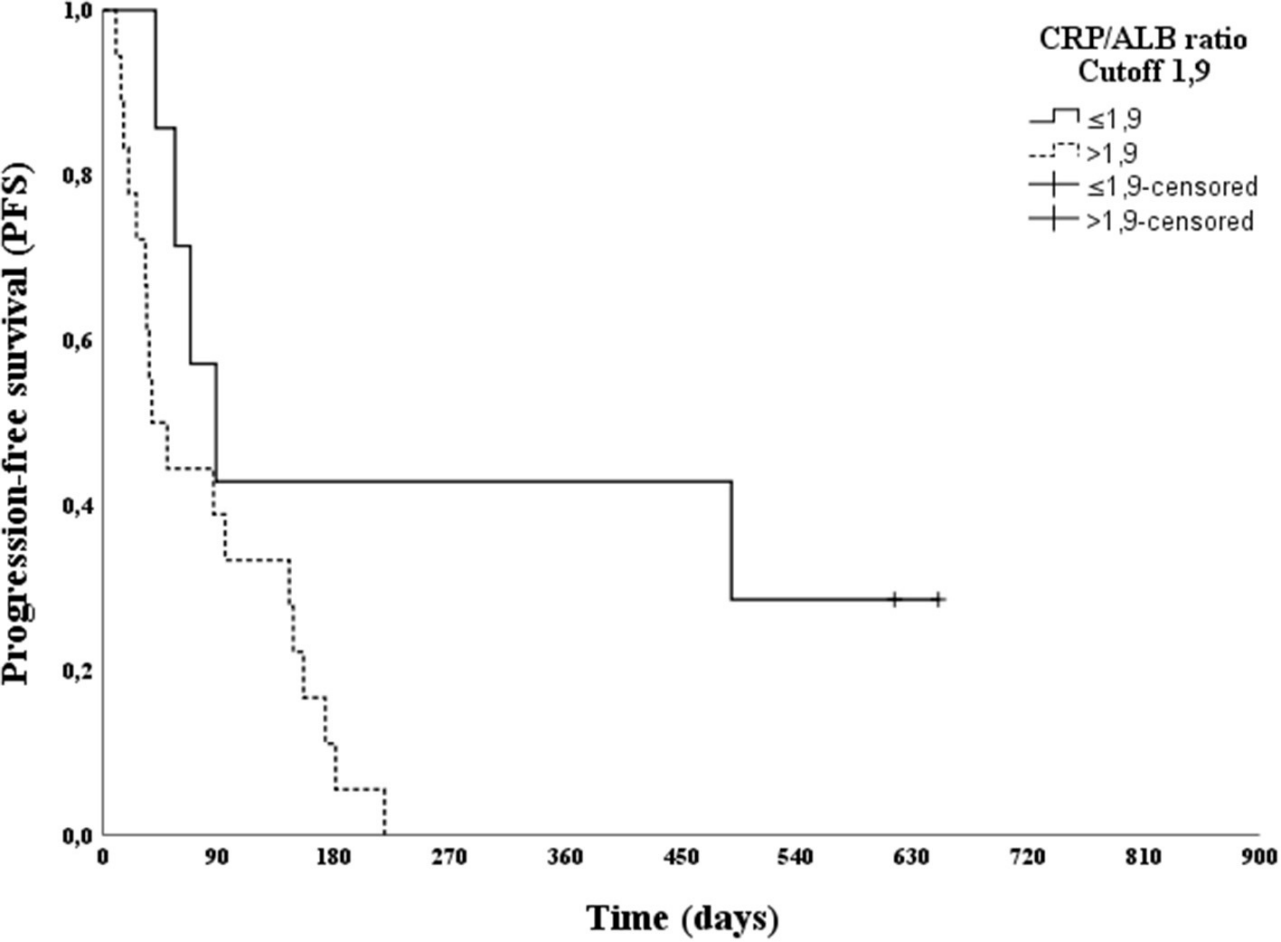
Kaplan–Meier progression-free survival (PFS) curve according to the C-reactive protein/Albumin (CRP/ALB) ratio cut-off point (1.9) in 25 canine patients with oral melanoma.

**Figure 3:**
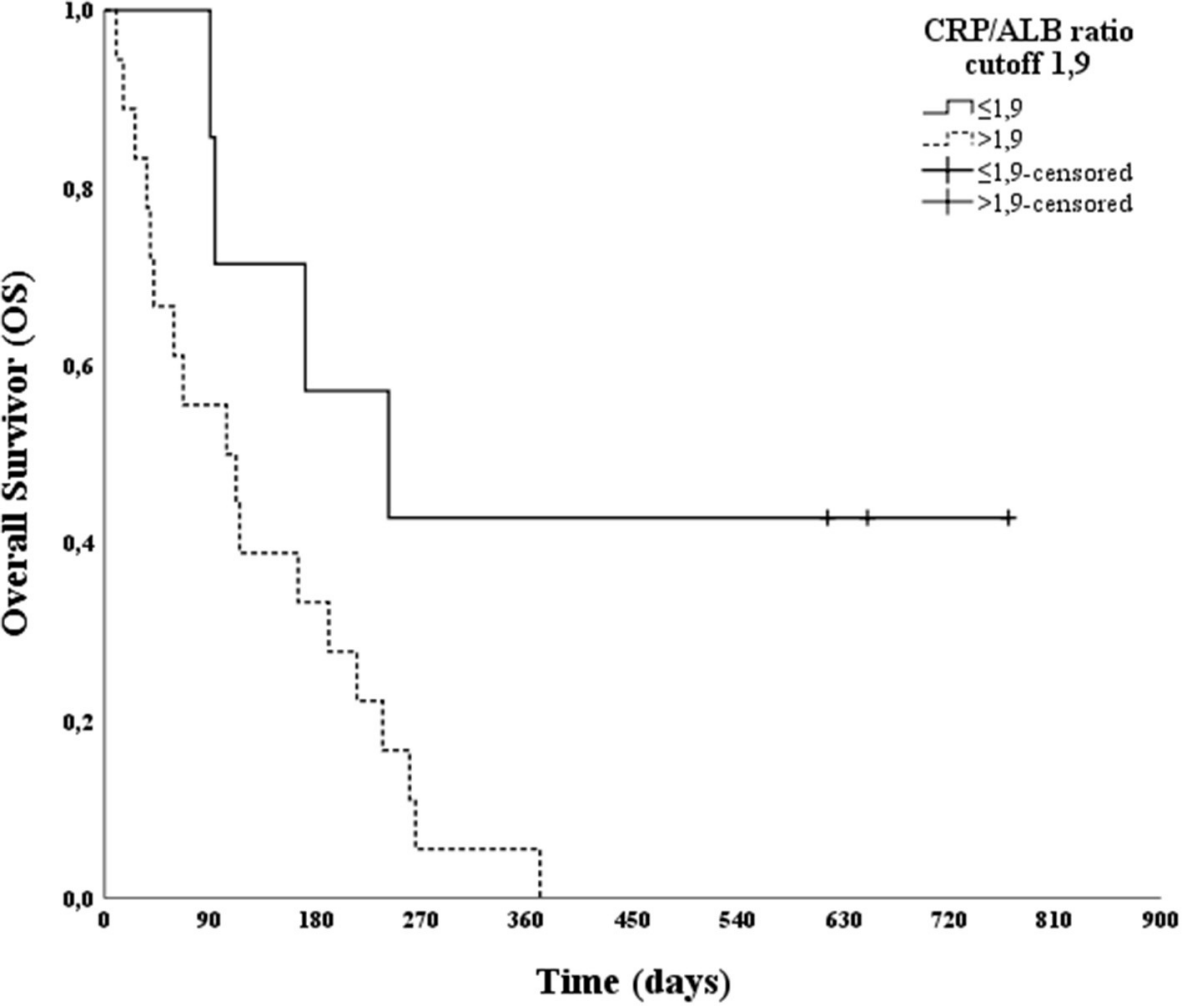
Kaplan–Meier overall survival (OS) curve according to the C-reactive protein/Albumin (CRP/ALB) ratio cut-off point (1.9) in 25 canine patients with oral melanoma.

The effect of a CRP/ALB ratio cut-off of 1.9 on PFS and OS was evaluated using a log-rank test. A statistically significant difference was found between patients with CRP/ALB ratios greater than or less than 1.9 (*p* = 0.036 for PFS and *p* = 0.023 for OS). Moreover, Kaplan–Meier curves were plotted (Figures 2, 3).

After treatment, 23 of the 25 patients experienced tumor recurrence, with 14 (61%) having recurrence at the tumor site and nine (39%) having metastases. Of the nine patients with metastasis, 6 (67%) had metastases to the lungs, while three (33%) had metastases to the regional lymph nodes.

## Discussion

The tumor microenvironment is characterized by persistent inflammation. In patients with solid tumors, inflammatory indices, LDH, CRP, albumin, and the frequency of immune circulating cells (lymphocytes and myeloid subsets) are inflammation-based prognostic scoring systems that reflect cell-mediated immunity and systemic inflammation (39). However, the utility of these reliable markers of the immune profile and systemic inflammation in preoperative evaluation for estimating prognosis and assessing combinatorial therapy outcomes in dogs with oral melanoma has not been verified. In the present study, all of these factors were subjected to univariate analyses, which revealed that the CRP/ALB ratio and Treg lymphocyte frequency were associated with PFS and OS. Therefore, these were added to a second, multivariable analysis, which showed that, in addition to the clinical stage, the CRP/ALB ratio and Treg frequency were associated with PFS and OS. The ROC curve showed that both parameters were accurate; however, only the CRP/ALB ratio showed an adequate AUC that made it possible to establish a cut-off value. Although the sensitivity was equal for different specificities, the derived cut-off point of 1.9 was the lowest value capable of distinguishing between patients with better and worse prognoses.

Our results showed that patients with CRP/ALB > 1.9 before tumor removal had shorter PFS and OS. Therefore, even with the small number of patients and the heterogeneity of our study group, the results showed that the CRP/ALB ratio is a promising prognostic tool for clinical staging in dogs with oral melanoma. In addition to being easily applicable in veterinary clinical routine, the levels of CRP and albumin are easily accessible outside the scope of research. In human medicine, several studies have recently shown the prognostic potential of the CRP/ALB ratio as a prognostic factor in cancer patients with different types of solid tumors (14, 16, 17, 19). However, in veterinary medicine, only one study has evaluated the importance of high values of this ratio in terms of prognosis in dogs with acute pancreatitis (40). The CRP/ALB ratio effectively reflects the patient's inflammatory status, as it assesses the increase in CRP (a positive acute-phase protein) and the concomitant decrease in albumin (a negative acute-phase protein).

Peripheral blood is a non-invasive source for exploring potential biomarkers. The other NLR, PLR, MLR, and SII indices showed no association with PFS and OS. The number of patients and group heterogeneity probably influenced these results, because, in human cancer patients, high values of these indices were associated with a lower survival rate and a lower treatment response rate in several types of tumors. In human patients, NRL ranges from 2 to 5 for different types of tumors, while in veterinary medicine, the NRL in patients with high-grade mast cell tumors, high-grade sarcoma, and oral cavity neoplasms was > 5.6, > 4.5, and > 8.56, respectively (24–26). The median NRL in the study group was 5.3, which is close to the NRL of dogs with mast cell tumors and high-grade sarcomas, and slightly lower than that of dogs with tumors in the oral cavity. The NRL of 5.3 was higher than that reported in the study by Rejec et al., which reported that healthy dogs and dogs with periodontal disease had an NRL of 2.7 and 4, respectively, and showed an increasing trend in more advanced stage patients, as the median NRL in patients with stages II, III, and IV was 5, 5.1, and 9.3, respectively (26). Rejec et al. also found a PLR of 145 and 290 in healthy dogs and those with oral cavity neoplasia, respectively. Even though the difference did not reach statistical significance, there was a tendency for PLR to be increased in dogs with cancer, a result similar to that of our study, which found a PLR of 250 (26). MLR and SII have not been studied in dogs, although high values have been associated with a worse prognosis in human patients with cancer (15). An important issue that was not evaluated in the present study is the influence of age, periodontal disease, and obesity on these inflammatory indices. Patients with obesity or those with concomitant periodontal disease were not excluded, and the median age of the patients in the group was 13 years. This study aimed to evaluate these indices considering their clinical applicability, since real-life patients with oral melanoma are older, obese, and have some degree of periodontal disease.

Measurement of LDH is part of the clinical staging of cancer in human patients with cutaneous melanoma. In particular, LDH levels are high among those with stage IV disease and who have a shorter OS (31). In our study, we found no evidence of association of LDH with PFS and OS, and the median LDH level was 762 IU/L. This value was higher than that reported by other studies, in which dogs with neoplasms had higher LDH levels than did healthy dogs. Marconato et al. studied dogs with different types of neoplasia and reported that the level of LDH was 341 UI/L in dogs with cancer, as compared to 142 UI/L in healthy dogs. Campos et al. studied dogs with mammary tumors and reported that the median LDH level was 414 UI/L in dogs with tumors, as compared to 201 UI/L in healthy dogs (33, 34). Zanatta et al. found a median LDH of 342 UI/L in healthy dogs and a value between 893 and 1,433 UI/L in dogs with multicentric lymphoma at different stages of therapy (32).

We also evaluated the frequency of circulating CD4^+^, CD8^+^, the CD4/CD8 ratio, Tregs, (M)-MDSCs, and (PMN)-MDSCs. There was no correlation of PFS and OS with the frequency of CD4^+^, CD8^+^, the CD4/CD8 ratio, and MDSCs. The frequency of CD4^+^ lymphocytes in dogs with melanoma in the present study was 43%, which was consistent with the prevalence in published studies of dogs with various types of cancer, in that the frequency of CD4^+^ ranged from 32 to 51% and did not differ markedly from the frequency in healthy dogs in these studies, which ranged from 35 to 38% (27–30). The same trend was observed with the frequency of CD8^+^ lymphocytes, which was 26% in our study (within the values found for cancer patients, which varied from 13 to 31%), but was not markedly different from the values found for healthy dogs, which varied between 20 and 26% in other studies (27–30). In our study, the CD4/CD8 ratio found was 1.5 in dogs with melanoma, close to the value of 1.6 found by Estrela-Lima et al. in dogs with metastatic malignant breast cancer and that of 1.43 found by Mucha et al. in dogs with various neoplasms (29, 30).

A higher frequency of Treg lymphocytes was associated with worse PFS and OS. Other studies have shown a trend toward an increase in Treg lymphocytes in dogs with different neoplasms and a more accentuated increase in dogs with metastatic disease (41–43). Tominaga et al. studied dogs with oral melanoma and found a 10% higher frequency of circulating Tregs than the 2.7% value found in our study (43). Other studies in dogs with different types of neoplasms found that Treg lymphocyte frequencies ranged from 5.2 to 13% in dogs with neoplasms and from 0.64 to 4.3% in healthy dogs. The differences across studies can be explained by the different methodologies used and the low number of patients. There may be a need for studies with a larger number of healthy dogs and dogs with cancer to establish a reliable reference value. Despite the increased frequency of Treg lymphocytes in our study, which may be used as a prognostic tool in future, this parameter is not yet accessible in clinical practice, but is used in research.

We found a total MDSC frequency of 22% in patients with melanoma, which is consistent with the findings of other studies that used similar methodologies to evaluate MSDCs, although there was no correlation of MDSC with PFS and OS (30, 44). Goulart et al. found a frequency of 36% in dogs with malignant neoplasms with metastases and 10% in healthy dogs (44). Mucha et al. reported an MDSC frequency of 18% in dogs with neoplasms with metastases, and 0.25% in healthy dogs (30). Other studies that characterized the subpopulations of (M)-MDSCs and (PMN)-MDSCs used different methodologies, such as different antibodies and types of samples, including whole blood or PMBCs, and found different proportions of these cell populations in the peripheral blood of dogs with cancer (45, 46). As there are no standardized methodologies, we cannot compare the findings of the current study with those of previously published studies. Our study showed that the frequency of the MSDC population found in dogs with oral melanoma was higher than that found in healthy dogs, following the same trend seen in human patients with cancer, who have a high frequency of these immunosuppressive cells in the peripheral blood (20, 30, 44).

In this study, we also sought to assess MC and/or immune lysate vaccine plus BCG could improve PFS and OS, but no statistically significant differences were observed between the groups with or without MC added to the tumor lysate vaccine. In addition to its antiangiogenic effect, MC has an immunomodulatory effect by reducing local and circulating immunosuppressive cells, such as Tregs, (M)-MDSCs, and (PMN)-MDSCs (47, 48). This effect was not observed in the study group, and we believe that the sample size studied was small, especially in the more advanced stage patients (only four patients with stage IV). Furthermore, the increased frequency of Tregs in our study showed that the immune profile of our patients reflected dysfunction or progressive loss of function of T cells in the tumor microenvironment.

We emphasize that in our clinical routine, most dogs diagnosed with oral melanoma were in advanced stages of the disease, given that this condition is typically diagnosed late. Another important consideration for the patients included in the study was the type of surgery performed, as most patients underwent surgery in which the tumor was not completely removed. This is due to the refusal of owners to perform major or mutilating surgeries on older dogs and refusal of using conventional chemotherapy due to the higher incidence of side effects. These patients were included in the study to receive the tumor lysate vaccine plus BCG, with or without MC, which has a lower incidence of side effects. In Brazil, electrochemotherapy has been widely used to eliminate tumor cells in the surgical bed. Radiotherapy is another modality available for local melanoma control; however, it is rarely used in Brazil due to the high cost of the procedure. These characteristics of our study group, i.e., local control of the disease (incomplete surgery with electrochemotherapy) and advanced stage (no patients in stage I and 56% patients in stage III), explain the shorter PFS and OS time in our study than in other studies conducted among dogs with oral melanoma. In our study, the median PFS and OS were 181 and 265 days for stage II, 40 and 101 days for stage III, and 38 and 68 days for stage IV, respectively, which are lower than those of other studies that only performed surgery in patients and obtained OS times of 511–874 days for stage I; 160–818 days for stage II, and 160–818 days for stage III disease (49, 50).

Studies in dogs with oral melanoma have determined that the type of surgery performed, and clinical stage are prognostic factors, since surgeries with wide surgical margins and patients with less advanced stages have a longer OS (50). However, there were no statistically significant differences in the type of surgery performed, as most patients (76%) underwent incomplete surgery, explaining the large local recurrence rate of 61%. There was a statistically significant difference in PFS and OS according to disease stage: 181 and 265 days for stage II, 40 and 101 days for stage III, and 38 and 68 days for stage IV. In the multivariable analysis, the clinical staging was added to the CRP/ALB ratio and Treg frequency as an adjustment as a well-established prognostic factor (5, 37, 50).

Surgery remains a key therapy in veterinary medical oncology for canine oral melanoma; however, new approaches have been proposed. The search for combinatorial therapies, such as debulking the tumor mass (complete or incomplete surgery), treatments with antineovasculogenic effects (MC), and potentiated immune responses (cancer vaccine strategies), expands the potential for more effective therapy for canine oral melanoma, a neoplasm of which the growth and invasion is difficult to control.

In conclusion, we did not find a better response to the vaccine in canine oral melanoma patients who received MC. In addition, we studied several inflammatory indices and the frequency of circulating cells to evaluate the inflammatory and immunological status of dogs with oral melanoma to predict the response to treatment and prognosis. The purpose of obtaining this information was to improve treatment planning. In addition, some of these parameters can be easily applied in routine clinical practice. The most promising of these are the CRP/ALB ratio and Tregs frequency. Nevertheless, the study had limitations due to the low number of animals and heterogeneity of the group in terms of clinical staging and forms of local control.

## Data Availability Statement

The raw data supporting the conclusions of this article will be made available by the authors, without undue reservation.

## Ethics Statement

The animal study was reviewed and approved by Ethics Committee on Animal Use (CEUA/FMVZ-USP) #3716290817. Written informed consent was obtained from the owners for the participation of their animals in this study.

## Author Contributions

JSG divulgated the study, received samples, and vaccine production, trained veterinarians, collected and compiled clinical data, performed blood analyses, analyzed the results, and wrote and edited the manuscript. VN received samples, vaccine production, and performed blood analyses. RL curated the data, performed the statistical analysis, and reviewed the results. CM supervised the study and acted as the study coordinator, acquired funding, and wrote and revised the manuscript. All authors have read and agreed to the published version of the manuscript.

## Funding

The authors are grateful to Fundação de Amparo à Pesquisa do Estado de São Paulo (FAPESP), process n° 2020/01004-3, and PhD scholarship granted by Brazilian Funding Institution CAPES.

## Conflict of Interest

The authors declare that the research was conducted in the absence of any commercial or financial relationships that could be construed as a potential conflict of interest.

## Publisher's Note

All claims expressed in this article are solely those of the authors and do not necessarily represent those of their affiliated organizations, or those of the publisher, the editors and the reviewers. Any product that may be evaluated in this article, or claim that may be made by its manufacturer, is not guaranteed or endorsed by the publisher.
