# Supplementary material for: Association of Systemic Inflammatory and Immune Indices With Survival in Canine Patients With Oral Melanoma, Treated With Experimental Immunotherapy Alone or Experimental Immunotherapy Plus Metronomic Chemotherapy
Source: Front Vet Sci. 2022 Jul 6;9:888411. doi: 10.3389/fvets.2022.888411 (PMC9296851; doi:10.3389/fvets.2022.888411)
Supplement: Supplementary file 1 [file Table_1.docx]

Supplementary Material

# Tumor lysate vaccine with Bacillus Calmette-Guerin (BCG)

After surgical excision (complete or incomplete), tumor samples were transferred to the laboratory within 24 h in a glucose solution or RPMI-1640 medium containing antibiotics under refrigeration to prepare the tumor lysate vaccine with BCG. Sample processing was performed in a laminar flow cabinet (sterile environment). The tumor was fragmented and digested with collagenase type II under agitation for 90 min in a water bath at 37 °C. The resulting solution was filtered using a 70-micron nylon filter, and cell viability was evaluated using trypan blue. Tumor cells were counted in Neubauer chambers, and each vaccine vial contained 3 × 10^6^–9 × 10^6^ cells in a final volume of 1 mL. The vials were stored at -80 °C and prepared within 1–6 h before application. The vaccine finalization process consisted of three hot (water at 100 °C) and cold (liquid nitrogen) cycles for 20 s. Subsequently, BCG (Imuno BCG^®^; the number of live bacilli greater than 2 × 10^6^ CFU/mg BCG, Fundação Ataulpho de Paiva, Rio de Janeiro, BRA) was added, and the vaccine was refrigerated until application. The BCG added to the first, second, and third doses was 160 µg, 80 µg, and 40 µg, respectively.
